# Sentinel Lymph Node Staging in Early-Stage Cervical Cancer: A Comprehensive Review

**DOI:** 10.3390/jcm13010027

**Published:** 2023-12-20

**Authors:** Chrysoula Margioula-Siarkou, Aristarchos Almperis, Giuseppe Gullo, Emmanouela-Aliki Almperi, Georgia Margioula-Siarkou, Eleni Nixarlidou, Konstantina Mponiou, Pavlos Papakotoulas, Chrysanthi Sardeli, Frederic Guyon, Konstantinos Dinas, Stamatios Petousis

**Affiliations:** 1 2nd Department of Obstetrics and Gynaecology, Aristotle University of Thessaloniki, 541 24 Thessaloniki, Greece; 2 Department of Obstetrics and Gynaecology, University of Palermo, 90133 Palermo, Italy; 3 Radiation Oncology Unit, Theageneio Anticancer Hospital, 546 39 Thessaloniki, Greece; 4 1st Medical Oncology Unit, Theageneio Anticancer Hospital, 546 39 Thessaloniki, Greece; 5 Department of Pharmacology & Clinical Pharmacology, School of Medicine, Aristotle University of Thessaloniki, 541 24 Thessaloniki, Greece; 6 Gynaecologic Oncology Unit, Institut Bergonie, 33000 Bordeaux, France

**Keywords:** lymph nodes, lymphadenectomy, diagnostic imaging, survival analysis

## Abstract

Cervical cancer (CC) continues to be a significant global public health concern, even with preventive measures in place. In women with early-stage CC, the status of lymph nodes is of paramount importance, not only for the final prognosis but also for determining the best therapeutic strategy. According to main international guidelines, pelvic full lymphadenectomy (PLND) is recommended for lymph node staging. However, in these early stages of CC, sentinel lymph node biopsy (SLNB) has emerged as a precise technique for evaluating lymph node involvement, improving its morbidity profile. We performed a literature review through PubMed articles about progress on the application of SLNB in women with early-stage CC focusing on the comparison with PET/CT and PLND in terms of oncological outcomes and diagnostic accuracy. While the superiority of SLNB is clear compared to radiologic modalities, it demonstrates no clear oncologic inferiority over PLND, given the higher detection rate of positive lymph nodes and predominance of no lymph node recurrences. However, due to a lack of prospective evidence, particularly concerning long-term oncological safety, SLNB is not the current gold standard. With careful patient selection and adherence to straightforward protocols, a low false-negative rate can be ensured. The aim of the ongoing prospective trials is to address these issues.

## 1. Introduction

Cervical cancer (CC) remains a significant health concern, as it still ranks as the second most common cancer among women and the third leading cause of cancer-related deaths in females [1]. The burden of this cancer is particularly high in developing countries due to limited screening programs. Strategies to mitigate its impact include patient education, changes in sexual behavior, and the introduction of HPV vaccination, especially considering that over 54% of patients diagnosed are under 50 years of age [2]. The oncologic outcomes of cervical cancer are widely dependent on the stage at diagnosis as overall survival rates are over 85–90% for early-stage disease, in contrary with the poor results observed for high-stage disease [3].

In the context of early-stage CC, the assessment of lymph node status holds utmost significance, as it plays a pivotal role in selecting the most appropriate treatment approach and significantly influences prognostic outcomes. The presence of lymph node metastasis significantly affects prognosis, with a drop in five-year Disease-Free Survival (DFS) from 88% to 57% [4]. As reported by the FIGO classification, early-stage CC includes FIGO stages IA1 with lymphovascular space invasion (LVSI), IA2, IB1, IB2, and IIA1. In these patients, currently, radical hysterectomy (RH) with bilateral pelvic lymphadenectomy is considered the choice of treatment. According to European and American guidelines, sentinel lymph node biopsy (SLNB) without additional pelvic lymphadenectomy (PLND) is acceptable for FIGO IA1 with LVSI and IA2 stages. However, for the IB1 stage, SLNB alone is not recommended without systematic PLND for lymph node staging except in the context of prospective clinical trials (as per ESGO/ESTRO/ESP guidelines), although the 2019 NCCN guidelines suggest considering SLNB biopsy for these cases [5,6]. PLND is mainly performed for staging reasons as it may tailor the decision to administer adjuvant radiotherapy, thereby achieving effective loco-regional disease control.

The size of metastatic lymph nodes is typically quite small, with dimensions smaller than 2 mm observed in 22% to 60% of patients with positive nodes [7,8,9]. Since most metastatic nodes measure less than 10 mm [10], both pelvic MRI and PET-CT tend to exhibit limited sensitivity and struggle to detect patients with positive lymph nodes [11]. However, the percentage of positive lymph node involvement in primary CC is estimated to be 15–20%, leading to 80% of patients undergoing PLND without benefit and resulting in associated complications, including an extended operative duration, increased blood loss, postoperative infections, the development of lymphocysts, and the occurrence of lower limb lymphedema [9,12,13]. Even though PLND is performed, it is worth noting that 10% to 15% of patients initially categorized as N0 (lymph node-negative) may still encounter cancer recurrences in the lymphatic region. This occurrence can be attributed to the presence of unconventional or atypical lymphatic drainage pathways [14].

In recent years, there has been a shift towards tailoring treatment, with increasing interest in less radical surgery for low-risk pathologic features. Sentinel Lymph node biopsy (SLNB) has been proposed to detect nodal invasion due to its acceptable effectiveness and improved morbidity profile [13]. Mapping studies have demonstrated that roughly 10% of sentinel nodes are situated in regions not universally encompassed within a conventional pelvic lymphadenectomy [15]. In the FIRES trial, it was observed that 17% of patients with node-positive disease exhibited disease that extended beyond the typical or traditional locations, with the most common sites of involvement being the presacral space, internal iliac region, and parametrial regions [16].

The main objective of this review is to summarize the current evidence on whether surgical staging with sentinel node or lymphadenectomy or imaging examinations is the optimal way to assess nodal status for early-stage cervical cancer patients, both in terms of diagnostic accuracy and final survival outcomes.

## 2. Materials and Methods

This is a comprehensive review of the literature aiming to summarize the current evidence regarding the diagnostic accuracy and survival outcomes of SLNB versus radiographic evaluation of nodal status and systematic lymphadenectomy in early-stage CC.

### 2.1. Searching Strategy

MEDLINE, UpToDate, and PubMed electronic databases were searched up to July 2023 for relevant published articles discussing the application of surgical and imaging nodal staging in early-stage CC. The searching strategy was formed by combining the MeSH terms and keywords “cervical cancer”, “Sentinel Lymph Node”, “Sentinel Lymph Node Biopsy”, “lymphadenectomy”, “surgical staging”, and “imaging staging”, with the help of the Boolean operators “or” and “and”.

### 2.2. Eligibility Criteria

Articles comparing SLNB staging with PLND and/or imaging staging that reported on survival outcomes of patients were mainly set in the scope of this present comprehensive review. Furthermore, studies indicating diagnostic accuracy in terms of false-positive and false-negative rates (FPR, FNR), sensitivity, and specificity regarding all staging modalities based on final pathology outcome were also eligible for inclusion. Additionally, studies addressing the impact of low-volume disease (micrometastases and isolated tumor cells) in the sentinel node on the survival outcomes or recurrence risk were also considered eligible. Regarding the types of studies, prospective and retrospective randomized control trials (RCTs), cohort studies, case–control studies, and case series studies, as well as systematic review articles and meta-analyses, were considered eligible to report, while case reports and published guidelines were also evaluated and reviewed for their content in order to extract relative data and information. References of included studies would additionally be cross-referenced to find additional publications eligible for inclusion in our review.

### 2.3. Exclusion Criteria

There was no exclusion criterion regarding the type of surgery (laparotomy, laparoscopy, robotic surgery) as well as regarding the imaging modality used (MRI, CT, or PET-CT). Studies would be excluded in cases where they lacked information on case numbers, FIGO stage, lymph node metastasis, and injection techniques (blue dye, radiocolloid tracers, both, or indocyanine green). Animal studies, abstract- or protocol-only publications, and video reports would also be excluded. Studies of patients with non-cervical malignancies or studies where radiologic-assisted biopsies were used to assess nodal status were also excluded. Finally, non-English articles and published abstracts without full-text manuscripts were all excluded from this review.

### 2.4. Study Selection Process and Results Organization

All studies identified from the search strategy were imported in a reference management software (Zotero 6.0.30) for elimination of duplicate data and further assessment. All identified studies were screened by two of the authors based on their full-text manuscript, while articles irrelevant to the objective of the present study were excluded. The eligibility of retrieved articles was independently determined by 2 reviewers (S.P., A.A.). Finally, at the end of evaluation process, the included articles were divided into 3 main categories: (a) articles comparing staging SLNB with imaging staging, (b) articles comparing staging SLNB with systematic PLND, and (c) articles reporting on oncological outcomes in patients with low-volume disease staged with SLNB. Moreover, the included articles in each category were additionally subdivided based on the type of study in the following four categories: (a) prospective cohorts, (b) RCTs, (c) retrospective cohort studies and case series, and (d) systematic reviews and meta-analysis articles.

## 3. Results

### Study Selection

Electronic searches and complementary hand-searching retrieved 433 articles. After the review of titles and abstracts, 390 articles were excluded and 43 studies were assessed for eligibility based on their full-text manuscript. There were finally 10 articles meeting the inclusion criteria. Specifically, two retrospective observational studies [17,18], one prospective [19], and one systematic review and meta-analysis [20] reported the higher diagnostic accuracy of SLNB vs. imaging staging. The SLN mapping method appears to perform extremely well both in cases with tumor < 20 mm and >20 mm, where the PET/CT seems to have limited diagnostic value. However, the combination of PET/CT with SLNB, in patients with tumor ≤ 40 mm, could represent a “safety net” to avoid overlooking metastatic lymph nodes. Overall, SLNB, when adhering to the algorithm, offers higher negative predictive value (NPV), specificity, and sensitivity compared to imaging staging. Regarding the survival outcomes of SLNB vs. PLNB, no clear inferiority of the former was demonstrated by a systematic review and meta-analysis [21], while one systematic review [13] was retrieved that showed the high diagnostic accuracy in terms of NPV and sensitivity. Additionally, regarding the impact of low-volume disease, two retrospective cohort studies showed conflicting results, with micrometastases (MICs) being associated with increased and decreased survival rates [22,23]. Another retrospective case–control study indicated MICs as an independent risk factor for recurrence [24]. Lastly, one prospective cohort study demonstrated no impact on survival outcomes [3].

## 4. Discussion

The current evidence suggests that surgical staging is the gold-standard method for nodal evaluation in early-stage CC, while systematic pelvic lymphadenectomy has been the standard of care in the nodal status assessment.

### 4.1. Procedure of Sentinel Lymph Node Biopsy

The sentinel lymph node (SLN) is identified as the initial node that receives drainage from a solid tumor and serves as an indicator of the status of nearby and subsequent nodes. This principle is applicable to tumors that follow a specific path of lymphatic drainage and exhibit a low likelihood of involving nodes. The focused extraction of these SLNs aims to verify the absence of tumor spread through the lymphatic system in the primary draining node. Consequently, it helps predict the lack of distant lymphatic metastases in secondary nodes. The SLNB, a precise sampling method, is intended to yield equivalent diagnostic insights as a pelvic lymphadenectomy (pN0 or pN1), while causing fewer complications and maintaining oncological outcomes intact. The number of lymph nodes removed during SLNB is typically limited (usually 1–3 nodes) as the focus is on identifying and examining the specific sentinel nodes for the presence of cancer cells. The aim is to minimize the invasiveness of the procedure while still obtaining crucial diagnostic information. Detected SLNs are removed selectively and sent for frozen section examination (FSE) after ex vivo palpation. Current guidelines suggest submitting SLNs to FSE to decide if a hysterectomy is to be pursued during the same surgical intervention. The time required for this analysis can range from approximately 20 min to 1 h, depending on the number of nodes [5].

The ESGO guidelines propose various methods for SLN mapping. One approach involves using a combination of blue dye (such as patent blue, isosulfan blue, or lymphazurin) along with radiocolloid Technetium 99m (99mTc). Alternatively, fluorescent dye alone, such as indocyanine green (ICG), can be utilized. It is recommended to avoid using blue dye alone, while combining it with isotopes may increase the sensitivity in detecting SLNs. During SLN mapping, injections of the tracing agents are administered submucosally at specific depths and points (into four quadrants of the cervix at 0, 3, 6, and 9 o’clock) using a 25-gauge needle. The advantages of blue dye are the low cost of the method because no additional equipment is required besides the dye while the main drawbacks seem to be the allergic reactions seen in less than 1% of the patients and the staining that it causes in the operative field, resulting in difficulties in SLN mapping [25]. In the last years, indocyanine green has been instituted in the field of SLNs; however, its superiority compared to 99mTc and its combination with blue dye has been challenged in a previous meta-analysis [26]. In the FILM trial that assessed the difference in the ability of SLN detection by different tracers, indocyanine green confirmed its superiority over the use of isosulfan blue dye solely [27]. ICG necessitates a specific near-infrared detection system for visualization, while blue dye is visible to the naked eye and does not require specialized equipment. Tracer injection is ideally performed at the beginning of the surgical procedure, post-patient positioning, to minimize false-negative outcomes by accurately identifying the true SLN and avoiding non-SLNs.

There are a number of protocols with minor alterations that are followed and are described. In Papadia et al.’s study [17], throughout the study period, different tracers were used. From April 2008 until January 2011, a combination of Tc-99 m-Nanocoll and blue dye was injected 1 day prior to surgery, and planar SPECT/CT using an integrated system was performed 1 h after injection to preoperatively locate the SLNs. Additionally, patent blue was injected in the operating room after the induction of anesthesia. From January 2011 until December 2016, SLN mapping was performed with intraoperative injection of a solution of ICG. In Tanaka et al. [18], on the day before the operation, 2.0 mL of fluid containing 110MBq 99m-Technetium (99mTc)-labeled tin colloids was injected into the patient’s cervix. SLNs were detected after entering the retroperitoneal cavity. Radioactive lymph nodes were located using a gamma probe. IDC-stained lymph nodes were detected by direct inspection. ICG fluorescence-positive lymph nodes were detected using a color fluorescence camera. In Sponholtz et al., a similar method of injecting 2 mL of diluted ICG on each side of the cervix at 3 and 9 o’clock positions with 1 mL submucosally was used. Intraoperative detection involves exploring abdominal and pelvic cavities for stained and/or radioactive nodes using a gamma probe, guided by lymphoscintigraphy information. Notably, it is stressed that not all detected nodes (blue and/or hot SLN) should be removed; rather, only the first draining node in the channel pathway needs removal and labeling as the SLN. This step is crucial in avoiding confusion with non-SLNs, which may occur due to tracer migration beyond the true SLN. However, in cases where truly separate channels correspond to distinct lymphatic drainage pathways, additional SLNs may be sampled. Enlarged lymph nodes that raise suspicion of metastatic disease undergo frozen section analysis to confirm the presence of nodal metastasis. If an intraoperative identification of a metastatic lymph node occurs, the surgical procedure is halted. In this scenario, the patient is directed towards chemo-radiotherapy following the detection of metastasis in the lymph node.

In conclusion, the significance of precise tracer selection, optimal timing of injection, accurate identification, and selective removal of SLNs to enhance the accuracy of SLN mapping and avoid misinterpretation of non-SLNs is emphasized.

### 4.2. Sentinel Node vs. Imaging Staging

In the context of imaging management, magnetic resonance imaging (MRI) has consistently been regarded as the optimal choice for the primary tumor assessment [28,29]. Conversely, when it comes to evaluating lymph node involvement, positron emission tomography (PET) has emerged as the preferred option [20]. From the 10 studies included in this review, 4 demonstrated the superiority of the diagnostic accuracy of SLNB vs. PET/CT in early-stage patients.

Specifically, according to two retrospective observational studies and one prospective multicenter study, the NPV, sensitivity, and specificity ranged between 97 and 100%, 75 and 96.3%, and 94 and 100%, respectively, for the SLNB group. On the other hand, for the imaging group, the NPV, sensitivity, and specificity ranged between 74 and 88%, 8 and 68%, and 84 and 98%, respectively [17,18,19]. In concordance with these data, but also displaying an even more accountable level of evidence, in a systematic review and meta-analysis of 72 primary studies involving 5042 patients, Selman et al. [20] demonstrated that SLNB had a pooled positive likelihood ratio of 40.8 and a pooled negative likelihood ratio of 0.18 for the determination of lymph node status. For PET/CT, the corresponding ratios were significantly lower (15.3 and 0.27). Furthermore, PET/CT offers superior sensitivity and specificity rates (73% and 98%, respectively) in comparison with MRI, which yields sensitivity and specificity rates of 56% and 93%, while CT shows rates of 58% and 92%.

In conclusion, SLNB emerged as a tool with greater accuracy in determining lymph node status among women with primary CC in comparison with all other advanced imaging modalities, from which PET is regarded as the optimal choice. Table 1 presents an overview of the published studies reporting on the diagnostic accuracy of SLNB and FDG-PET/CT.

### 4.3. Sentinel Node vs. Systematic Lymphadenectomy

The use of SLN detection has gained popularity based on the concept that once lymph node involvement is confirmed, extensive lymph node dissection (PLND) does not offer a significant prognostic advantage, with the focus shifting towards adjuvant treatment. Thus, the central question is whether a complete lymph node dissection may have an additional therapeutic and survival impact.

A systematic review and meta-analysis was conducted to address this question, including four studies, of which one was prospective randomized multicentric study, while the remaining three were retrospective cohorts [21]. The meta-analysis encompassed 1952 patients of FIGO Stage IA1 to IIA, with 383 undergoing exclusively SLNB and 1569 undergoing PLND. However, it should be noted that in one cohort study with the largest number of patients (1188), the two groups differed in a wide range of enrollment time windows (2005–2015 for SLN vs. 1984–2005 for PLND), which may, hypothetically, be affected by technological advancement and may explain the difference in the results of these two methods, while the other three studies were well balanced in age and tumor characteristics, making the results comparable. Due to low heterogeneity of DFS (I2 = 33%; *p* = 0.21) and OS (I2 = 33%; *p* = 0.21), a fixed-effects model was applied for them. Over a 4.5-year period, the DFS rates varied between 85.1% and 93.8% for the SLN group and between 80.4% and 93.1% for the PLND group. There was no significant difference between these two groups (OR 1.04, 95% CI 0.66–1.66, *p* = 0.85). In contrast, the recurrence rate ranged from 3.6% to 11.5% for the SLN group, while the range was narrower, namely between 6.4% and 7.3%, for the PLND group. Unfortunately, only three out of the four comparative studies provided data on OS, involving 302 patients in the SLN group and 1351 in the PLND group. Interestingly, the analysis did not reveal any significant difference in OS benefits between the SLN and PLND groups.

Regarding the detection superiority in nodal staging, SLN mapping has displayed considerable accuracy. In retrospective series, the incidence of false-negative findings has been documented at less than 1%, with high sensitivity and NPV percentages of 96.4% (95% CI 79.8–99.8%) and of 99.3% (95% CI 95.6–100%), respectively [30,31]. Furthermore, a recent systematic review focusing on early-stage CC revealed an SLN metastasis prevalence of 21%. The sensitivity of SLN mapping in this context was notably high at 94%, accompanied by an NPV ranging from 91% to 100%. Impressively, the FNR was found to be as low as 1.5% [13]. These findings underline the efficacy and reliability of SLN mapping as an integral component in the management of early-stage CC.

### 4.4. Diagnostic Accuracy of SLNB—Impact of Low-Volume Disease

The National Comprehensive Cancer Network (NCCN) guidelines recommend ultrastaging of sentinel lymph nodes (SLNs) [6]. Ultrastaging involves meticulously examining SLNs with serial sectioning and immunohistochemistry for cytokeratin, particularly on hematoxylin and eosin (H&E) negative slides. This approach detects low-volume metastases that conventional lymphadenectomy might miss. Metastatic lymph nodes are categorized by size, macrometastases (MACs) (>2 mm), micrometastases (MICs) (0.2–2 mm), and isolated tumor cells (ITCs, <0.2 mm), as defined by the American Joint Committee on Cancer (AJCC) [32].

Routine lymphadenectomy typically does not undergo ultrastaging. When SLN ultrastaging is employed, it increases the likelihood of diagnosing stage IIIC disease due to the presence of MICs and isolated tumor cells [33]. Consequently, patients undergoing SLNB are more likely to be identified with metastasis and receive adjuvant therapy compared to those undergoing complete lymphadenectomy. A debate surrounds the significance of these low-volume metastases in SLN biopsy. They may either improve traditional staging by identifying previously overlooked disease or introduce “false positive” results if these small metastatic foci are clinically inconsequential.

While the clinical relevance of MACs and the indication of adjuvant treatment is clear, the impact of MICs and ITCs remains uncertain. Multiple retrospective studies have explored the impact of low-volume metastases, specifically MICs and ITCs, in cervical cancer, demonstrating mixed results. The largest among them was conducted by Cibula et al. [22] involving 645 patients and revealed an association between the presence of MACs or MICs and reduced OS. Additionally, Marchiolé et al. [24] identified MICs as an independent risk factor for recurrence. However, Zaal et al. [23] reported that when MICs were present, the OS improved with the dissection of more than 16 lymph nodes. Notably, ITCs did not exhibit prognostic relevance in their study.

Comparing these studies is challenging due to variations in methodology, particularly in ultrastaging techniques and patient selection. In an attempt to address these discrepancies, Guani et al. [3] conducted a prospective study assessing recurrence and survival in early-stage CC patients with MICs or ITCs. Surprisingly, they found no impact of MICs or ITCs on progression-free survival (PFS). However, the authors acknowledged that although the study was prospective, its relatively small sample size (139 patients) limited its ability to provide definitive answers. Small-scale studies suggest that it still remains a question whether low-volume disease may have limited clinical impact since long-term data are lacking. In conclusion, further research is necessary to establish the true clinical significance of low-volume metastases in cervical cancer progression.

Finally, there are currently three active prospective clinical trials being performed in order to evaluate the oncological consequences of SLNB in early-stage CC. These trials include the SENTIX trial (NCT02494063) [34], a prospective multicenter observational study focused on assessing the 2-year recurrence rate following solitary SLNB. The PHENIX trial (NCT02642471) [35] is a multicenter randomized controlled trial designed to compare oncological outcomes, with a specific focus on patients with SLN metastasis (evaluating 2-year DFS) and those without it (evaluating 3-year DFS). Lastly, the SENTICOL III trial [36] is a prospective multicenter randomized study with the primary aim of comparing 3-year DFS rates between two approaches: SLNB as a standalone procedure and SLNB in conjunction with PLND. These trials collectively contribute to advancing our understanding of SLN biopsy’s role in managing early-stage CC.

## 5. Limitations

The present article represents only a critical review trying in a systematic way to evaluate the current evidence on this issue. However, the retrieval of studies and interpretation of results rather led to specific conclusions, standing firmly in favor of the safety and effectiveness of SLNB in early-stage CC cases. To our knowledge, this is potentially the first review article trying to map all heterogeneous results mentioned regarding an issue for which profound methodological difficulties have not yet permitted the performance of a large RCT and potentially achieving this.

## 6. Implications for Practice and Future Research

No clear impact of surgical staging on survival outcomes has been demonstrated by the majority of the published evidence, even if imaging staging has low sensitivity and a high false-positive rate. As previously stated, this meta-analysis’s conclusions imply the need to have the definitive results of a prospective multicenter RCT, enrolling certified centers and physicians with skills in performing optimal surgical staging.

## 7. Main Conclusions

The present critical review demonstrated that the majority of the published evidence firmly stands in favor of the safety and effectiveness of SLNB over imaging staging, since SLN mapping presented a higher sensitivity and specificity rate compared with PET/CT. Furthermore, SLNB has emerged as a surgical strategy with comparable accuracy and reduced complications compared with full lymphadenectomy. However, there is no compelling evidence to abandon PLND in early-stage CC, and major international guidelines still recommend it. Further research based on a large multicenter prospective RCT could potentially lead to definitive conclusions and present an adequate level of evidence to answer this clinical question.

## Figures and Tables

**Table 1:** Overview of published studies reporting on the diagnostic accuracy of SLNB and FDG-PET/CT nodal status evaluation in early-stage cervical cancer. (NR: Not Reported).

Title	Type of Study	Number of Patients (n)	Negative Predictive Value Imaging vs. Surgical (95% CI)	Positive Predictive Value Imaging vs. Surgical (95% CI)	Sensitivity Imaging vs. Surgical (95% CI)	Specificity Imaging vs. Surgical (95% CI)	Pooled Positive Likelihood Ratio Imaging vs. Surgical (95% CI)	Pooled Negative Likelihood Ratio Imaging vs. Surgical (95% CI)
Papadia et al. [17]	Retrospective observational study	n = 60	88% (0.76–0.94) vs. 97% (0.88–0.99)	61% (0.47–0.73) vs. 100% (0.91–0.1)	68% (0.55–0.79) vs. 93% (0.82–0.98)	84% (0.71–0.91) vs. 100% (0.91–0.1)	NR	NR
Tanaka et al. [18]	Retrospective observational study	n = 48	88.2% vs. 100%	NR	8.3% vs. 75.0%	97.6% vs. 94.0%	NR	NR
Sponholtz et al. [19]	Prospective multicenter study	n = 245	73.9% (63.4–82.7) vs. 98.7% (93.0–100)	Imaging only 26.7% (7.8–55.1)	14.8% (4.2–33.7%) vs. 96.3% (81.0–99.9%)	Imaging only 85.5% (75.6–92.5%)	NR	NR
Selman et al. [20]	Systematic review and metanalysis	n = 5042	NR	NR	74.7% (63.3–84.0) vs. 91.4% (87.1–94.6)	97.6% (95.4–98.9) vs. 100% (99.6–100)	15.3 (7.9–29.6) vs. 40.8 (24.6–67.6)	0.27 (0.11–0.66) vs. 0.18 (0.14–0.24)
